# Carpophiline-ID: an interactive matrix-based key to the carpophiline sap beetles (Coleoptera, Nitidulidae) of Eastern North America

**DOI:** 10.3897/zookeys.1024.59467

**Published:** 2021-04-06

**Authors:** Courtney L. DiLorenzo, Gareth S. Powell, Andrew R. Cline, Joseph V. McHugh

**Affiliations:** 1 Department of Entomology, University of Georgia, 455 Biological Sciences Building Athens, GA 30602, USA University of Georgia Athens United States of America; 2 Department of Biology, Brigham Young University, Provo, UT 84602, USA Brigham Young University Provo United States of America; 3 Plant Pest Diagnostics Center, Department of Food & Agriculture, 3294 Meadowview Rd Sacramento, CA 95832, USA Plant Pest Diagnostics Center, Department of Food & Agriculture Sacramento United States of America

**Keywords:** Anatomy, characters, determinations, identification key, interactive key, matrix key, morphology, multi-entry key, taxonomy

## Abstract

Carpophiline-ID is presented, a matrix-based Lucid^TM^ key, for the adult stage of the known species of Carpophilinae (Coleoptera: Nitidulidae) of North America, east of the Mississippi River. An overview of the features and technical specifications used to build the key is provided. The list of terminal taxa used in the key represents the most current regional account for Carpophilinae, a beetle subfamily of agricultural and ecological importance. The value of matrix-based, free access keys for the identification of difficult taxa is discussed.

## Introduction

Matrix-based keys, such as Lucid keys, are often superior to traditional dichotomous keys. They allow users to follow different paths to a determination, use particular subsets of characters, use multi-state and non-traditional characters (e.g., biological, geographical, phenological, and genetic data), and allow creators to incorporate extensive supporting graphics to aid in identification (Penev et al. 2009, 2012; Cerretti et al. 2012). Lucid keys are structured around a data matrix of diagnostic characters scored for each taxon in the key. Identifications proceed by users selecting any character in the key and indicating the state observed for the specimen. The software eliminates taxa that do not match the selected criterion. This format makes the key “undirected”, allowing users to take multiple paths, skipping difficult, missing, or inapplicable characters to identify specimens. Since undirected keys work by eliminating taxa that do not match the character states observed for the subject, users may choose more than one “observed” state option if uncertain. Lucid provides a web hosting service, making these keys widely accessible for free to the scientific community and the general public. These advantages make Lucid^TM^ keys superior to traditional dichotomous keys, especially when dealing with difficult to identify specimens.

Sap beetles are represented by ~ 4,500 species in ~ 350 genera worldwide (Ślipiński et al. 2011). Of those, ~ 165 species in ~ 30 genera are known to occur in North America (Habeck 2002). East of the Mississippi River, the subfamily Carpophilinae is represented by four genera and 21 species, most of which are widely distributed. Carpophilinae are distinguished from other Nitidulidae in the Nearctic Region by the following combination of characters: Elytra short and apically truncate, not covering pygidium and one or two preceding tergites; terminal segment of labial palpi somewhat enlarged, shorter to slightly longer than wide, widely truncate at apex; antennal grooves often elongate, convergent posteriorly; elytra lacking sutural striae, elongate marginal setae present laterally, longitudinal carinae and longitudinal rows of setae, or punctures present (Habeck 2002).

Several species of Carpophilinae, especially those in *Nitops* Murray, feed on pollen as adults. Ongoing research continues to determine pollination efficacy by these beetles, especially for plants in the genus *Annona* L. (Magnoliales: Annonaceae) (George et al. 1989; Tsukada et al. 2008; Higuchi et al. 2014). Most carpophiline taxa are associated with ripe, rotting, or dried fruits and vegetables; however, some species have demonstrated the ability to damage healthy fruit and transmit bacterial pathogens, making them pests of agricultural commodity production (Leschen and Marris 2005). Currently, best management practices for Carpophilinae in stone fruits are field sanitation, harvesting fruits before fully ripe, and using trap and kill bait stations to reduce populations levels (Hossain et al. 2006; Bartelt and Hossain 2006). Trap and kill bait stations use lures containing pheromones to capture beetles. To be successful, it is important to correctly identify the species involved so the correct pheromones can be deployed. Due to the association with ripe and stored crops, many species are spread through international food trade. The ability for port and border inspectors to correctly identify detected carpophilines is paramount in preventing the entry of invasive exotic species at ports of entry.

Historical dichotomous keys to Carpophilinae of the USA are available (e.g., Parsons 1943; Connell 1977; Connell 1991). However, these keys exclude several eastern species and rely on difficult characters, limiting usefulness and applicability. In addition, high quality graphics illustrating each species and difficult characters are lacking from those works. To aid in accurate identification of this difficult and important group, a web-based Lucid key was developed for the Carpophilinae genera and species in eastern North America.

## Project description

### Taxonomic coverage

This key covers all Carpophilinae known to occur east of the Mississippi River in the USA and east of 90° longitude in Canada, including all four genera and 21 of the 34 species currently known to occur in America north of Mexico.

#### List of the terminal taxa included in the current version of the identification key (last update September 2020)

*Caplothoraxlugubris* (Murray, 1864); *Caplothoraxmelanopterus* (Erichson, 1843); *Caplothoraxsayi* (Parsons, 1943); *Carpophilusantiquus* Melsheimer, 1844; *Carpophilusbrachypterus* (Say, 1825); *Carpophiluscorticinus* Erichson, 1843; *Carpophilusdimidiatus* (Fabricius, 1792); *Carpophilusdiscoideus* (LeConte, 1858); *Carpophilusfumatus* Boheman, 1851; *Carpophilushemipterus* (Linnaeus, 1758); *Carpophilusmarginatus* Erichson, 1843; *Carpophilusmarginellus* Motschulsky, 1858; *Carpophilusmutilatus* Erichson, 1843; *Carpophilusnepos* Murray, 1864; *Carpophiluspilosellus* Motschulsky, 1858; *Carpophilustempestivus* Erichson, 1843; *Nitopscraigheadi* (Dobson, 1972); *Nitopsfloralis* (Erichson, 1843); *Nitopsophthalmicus* (Murray, 1864); *Nitopspallipennis* (Say, 1823); *Urophorushumeralis* (Fabricius, 1798).

#### Images of terminal taxa

For each species represented in the key, there is a minimum of one male dorsal and one male ventral habitus photograph. All specimens imaged were determined by the second and third authors (GSP & ARC). Photographs illustrating each character and all of the various states are provided in the key. All species-specific character images are included in the appropriate Species Fact Sheet, along with corresponding dorsal and ventral habitus images. These Species Fact Sheets can be accessed by hyperlinks provided for each species in the Entities section of the key.

### Characters used in the key

#### General features

Diagnostic characters in the key were derived from existing literature (Parsons 1943; Connell 1977; Connell 1991), study of specimens, and from museum specimen collection data. Published attributes of species were confirmed using specimens in the Smithsonian Institution National Museum of Natural History (**NMNH**), Illinois Natural History Survey Insect Collection (**INHS-INHSIC**), Florida State Collection of Arthropods (**FSCA**), and University of Georgia Collection of Arthropods (**UGCA**). Morphological terms used here follow those of Parsons (1943) and Connell (1977). An anatomical atlas is included in the Features section to aid non-specialists in interpreting characters used in the key.

The data matrix includes 41 anatomical, distributional, and ecological characters. These characters appear in the Features section, each with two to five possible character states. All features refer to either external adult anatomical structures that can be easily seen with a stereomicroscope, ecological details, or the locality where the specimen was collected. For length and ratio features, numeric ranges were derived from the literature (Parsons 1943; Connell 1977, 1991) and measurements taken from museum specimens. Morphological features are grouped by the following structures/regions: antenna, eye, pronotum, prothorax, mesothorax, elytra, metathorax, pygidium, and abdominal ventrites. This approach allows the user to quickly find characters of interest. Characters based on the distribution, ecology, and overall specimen appearance are grouped under the heading “general features.” Since some morphological features are only present in either the male or female, characters not relevant for a particular specimen can be excluded from consideration quickly by indicating the sex of the specimen. The key is intended for non-experts and is strongly image-based. For relative diagnostic characters (e.g., “dense” vs. “sparse”), users are expected to select the image of the character state that most closely matches what they observe on their specimen. As a result, they do not need to know the range of variation of that character within this taxon and do not need any special equipment other than a microscope to use the key.

#### List of characters used in the key

GENERAL: sex (male/female); length (mm); host association (cactus flowers/other hosts or habitats); geographic distribution (Northeast/Mid-Atlantic/Great Lakes Region/Mississippi Valley/Southeast); body convexity in lateral view (flattened/convex); body surface overall appearance (glossy/dull).

ANTENNA: antennal club shape (round/oval); antennomere coloration (abruptly darker at club/gradually darker towards club/unicolorous throughout).

EYE: ratio of eye width at widest point: intraocular distance at narrowest point (1:3 or less/between 1:4 and 1:9/1:10 or more).

PRONOTUM: pronotal disc setation length (long/not distinctly long); pronotal disc punctation density (dense/sparse/not conspicuously dense or sparse); pronotum coloration (black/dark brown/medium brown, light brown, or orange); pronotum posterior angles (broadly rounded, no distinct ‘corner’ created/squared due to extra anterior deflexion of lateral margin/nearly forming a 90-degree angle, not broadly rounded or squared off).

ELYTRA: elytral coloration (bearing pattern or markings/unicolorous); elytral color pattern (conspicuous yellowish humeral and apical patches/light humeral patches only/darker coloration near scutellum and apex/darker coloration near scutellum only/dark coloration near apex only); elytral coloration unicolorous (unicolorous and distinctly darker than pronotum and tergites/unicolorous and distinctly lighter than pronotum and tergites/unicolorous, similar to pronotum and tergites); shape of elytra apex (straight, squarely truncate/rounded, arching posteriorly).

PROTHORAX: ratio of prosternal process width at narrowest point between coxae to width at widest point posterior to coxae (less than 1:2/greater than 1:2); median longitudinal carina on prosternal process (present/absent).

MESOTHORAX: posterior rim of mesocoxal cavities (crenulate, not forming axillary space/smooth, not forming axillary space/smooth, forming small axillary space extending ~ ¼ posteriad along metepisternal suture/smooth, forming large axillary space extending ½ posteriad along metepisternal suture); mesosternal median longitudinal ridge (present/absent); mesosternal anterior impunctate edge along median longitudinal ridge (present/absent, bearing longitudinal ridge only); mesosternal impunctate area near center (present/absent, punctate throughout).

METATHORAX: male metathoracic tibial shape (abruptly dilated apically/gradually dilated apically); male metathoracic femur (bearing small toothlike projection on inner margin near trochanter/lacking a tooth-like projection near trochanter); metathoracic tibial spines along posterior margin (present, distinct/absent, not distinct).

PYGIDIUM: male pygidial lateral margin shape (visibly constricted/not constricted); male supplementary segment visibility in dorsal view (visible/not visible); female pygidium bearing large oval depression with vague anterior margin at apex (present/absent); female pygidium apical flexion (deflexed ventrally/upturned medially/not flexed upward or downward); female pygidium bearing weak median longitudinal ridge (present/absent); female pygidium bearing grooves along lateral margins (present/absent); female pygidium lateral margin shape (visibly constricted/not constricted, straight); female pygidium apical margin shape (pointed, acute/broadly rounded or truncated); female pygidial disc setation length (short, at most just able to reach base of nearest seta/medium to long, clearly able to overlap adjacent setae); female pygidial disc setal density (dense/sparse); female hypopygidium setation length (apical setae longer than other setae/apical setae not distinctly different in length from other setae).

ABDOMINAL VENTRITES: male 4^th^ ventrite setation (bearing many distinctly elongate setae medially at posterior margin/without any distinctly elongate setae); male 5^th^ ventrite setation compared to density anterior to supplementary segment (less dense or absent/not distinctly different density); male 5^th^ ventrite depression, shape and location (single undivided median circular depression with coarse punctation anterior to supplementary segment/single undivided median circular depression anterior to supplementary segment/single median rounded depression divided by horizontal ridge anterior to supplementary segment/pair of elongate oval depressions lateral and anterior to supplementary segment/without any depressed area anterior to supplementary segment); male setation on supplementary segment (setose with two distinctly longer setae/setae of approximately equal length); female hypopygidium setation length (apical setae longer than other setae/apical setae not distinctly different in length from other setae).

### Software technical specifications

**Application**: Lucid Builder 3.5 (https://www.lucidcentral.org, website provides technical specifications and features list)

**Key Version**: 1.0

**Requirements for use**: Java-enabled browser and internet connection

**License for use of the key**: Creative Commons Attribution License 4.0 (CC-BY), which permits unrestricted use, distribution, reproduction, and editing, provided the original author and source are credited.

**Web location**: https://site.caes.uga.edu/carpophiline-id/

## Data resources

The data underpinning the Lucid Key (Lucid Key files) reported in this paper are deposited in the Dryad Data Repository at https://doi.org/10.5061/dryad.h44j0zphq

### Website features

#### Species fact sheets

https://site.caes.uga.edu/carpophiline-id/taxon-fact-sheets/

All 21 species represented as entities in the key are figured with dorsal habitus, ventral habitus, and diagnostic character images. Each species fact sheet includes a diagnosis and summaries of the known biology and distribution, as well as references. Within the interactive key, these pages can be accessed through hyperlinks provided within each species entry.

#### Resources

https://site.caes.uga.edu/carpophiline-id/resources/

This section provides an anatomical atlas (also available within the key), a glossary of terminology, and diagnoses for the beetle family Nitidulidae and the subfamily Carpophilinae. The anatomical atlas illustrates all of the structures mentioned in the key on a dorsal and/or ventral habitus image of a male specimen of *Carpophilusmarginellus*. The glossary provides definitions of terms used in the key. Definitions were derived from Nichols (1989), Parsons (1943), and Connell (1977). The diagnostic pages provide lists of anatomical characters used to recognize beetles belonging to Nitidulidae and the subfamily Carpophilinae.

#### References


https://site.caes.uga.edu/carpophiline-id/references/


This section provides a list of useful references about Carpophilinae, building interactive keys, and making species fact sheets.

## Conclusions and future work

Since multi-access keys enable users to skip sex-specific, hard-to-view, and rarely available characters, additional, more difficult diagnostic features (e.g., male genitalic anatomy, features on immature stages, genetic markers, etc.) will be added as they become available. A comprehensive taxonomic revision of the Carpophilinae of North America is currently being conducted (by GSP). Upon completion, newly published information may be incorporated into the data matrix and species fact sheets to update the interactive key.

This key provides a user-friendly tool that will make species-level identifications of Carpophilinae possible for specialists and non-specialists. Additions and updates will be possible as new characters become available and taxonomic changes occur within the group. The key may also be expanded to include newly discovered species, or to extend geographic distributions to create a more inclusive tool.
